# Program impact and potential pitfalls of multi-purpose technologies (MPTs) for HIV prevention and contraception

**DOI:** 10.3389/frph.2023.1249979

**Published:** 2023-09-01

**Authors:** Mary H. Latka, Kristin Vahle, Kevin Li, Megan Gomes, Anita Dam

**Affiliations:** ^1^ Office of HIV/AIDS, Bureau of Global Health, United States Agency for International Development, Washington, DC, United States; ^2^ GHTASC, Credence LLC, Washington, DC, United States; ^3^ STAR, Public Health Institute, Washington, DC, United States; ^4^ Office of Population and Reproductive Health, Bureau of Global Health, United States Agency for International Development, Washington, DC, United States; ^5^ GHTASC, Public Health Institute, Washington, DC, United States

**Keywords:** multi-purpose technology, HIV prevention, contraception, unplanned pregnancy, use-effectiveness, method efficacy, adolescent girls, sub-Saharan Africa

## Abstract

The overlapping epidemics of HIV and unplanned pregnancy disproportionately affect adolescent girls and young women (AGYW) in sub-Saharan Africa. Prevailing dynamics driving benefits of any prevention method at the population level depend on: 1) population size, risk profile, and prevalence of method use, 2) method efficacy, and 3) method use-effectiveness. Adding a multi-purpose technology (MPT) to prevent HIV and pregnancy to this three-part equation results in scenarios that may enhance HIV population impact, even with methods that exhibit less than “perfect” method efficacy, by extending protection among existing users and attracting new users, resulting in greater population coverage. However, the interplay of epidemic drivers is complex and the greatest population benefit of such a MPT would be realized among those most at risk for HIV and pregnancy, and could be harmful if successful contraceptive users switch to a method with lower use–effectiveness. While MPTs are highly desired, and may offer considerable individual, population, and system-level public health benefits, there is no “magic bullet”, nor single prevention method–MPT or otherwise–that will end the HIV epidemic nor fully resolve unmet need for family planning. All methods have inherent tradeoffs and women have varied reproductive and HIV prevention needs across their life course. Key programmatic features to maximize the potential of MPTs include offering them among a range of safe and effective methods with comprehensive information about their features allowing women to make a fully-informed method choice. Programmatic follow-up should support consistent and correct use to maximize use-effectiveness, and then monitor for potential untoward effects.

## Introduction

Multipurpose prevention products (MPTs) under development include a range of delivery platforms such as rings, implants, injectables, films, enemas, and vaginal and rectal inserts for HIV, other sexually transmitted infections (STIs), and contraception (1,2). Currently, condoms are the only MPT available for protection against STIs, HIV, and pregnancy. Condom use is challenging, especially for adolescent girls and young women (AGYW) in sub-Saharan Africa (SSA) (3). Men's and women's desire for children, gender inequality, domestic violence, and stigma hinder agency for use. Warfare, corruption, poverty and competing morbidities create unstable backdrops for HIV prevention programming. Product development funding and product cost also present challenges. These prevailing societal norms and realities challenge introduction and use of any new prevention method. The majority of MPTs in development target HIV and pregnancy (1). Women's reproductive health and HIV are related, with HIV exacerbating the maternal mortality epidemic and mother-to-child transmission significantly contributing to the HIV epidemic in SSA (4). A biomedical MPT preventing HIV and pregnancy could have substantial health benefits for AGYW in SSA. The HIV epidemic disproportionately affects AGYW in SSA with 63% of incident infections occurring in females aged 15–24 (5). Further, across 30 SSA countries, the prevalence of unmet need for contraception is 27% among partnered AGYW aged 15–24, and 32% of pregnancies among women in SSA are unplanned (6,7).

A MPT for HIV and pregnancy–even one with less than perfect method efficacy for HIV prevention–holds potential for enhanced public health impact due to the interplay of three drivers of protection: 1) the population's size, risk profile, and method use prevalence; 2) method efficacy and; 3) method use-effectiveness. Adding MPTs for HIV and pregnancy to this three-part equation results in scenarios that may enhance HIV population impact. However, epidemic drivers are complex, and some MPT introduction scenarios for HIV and pregnancy may detract from net population benefit. The greatest potential population benefit and efficiencies for such a MPT would be realized among those most at risk for HIV and pregnancy.

## Three drivers of population benefit

**Figure 1:**
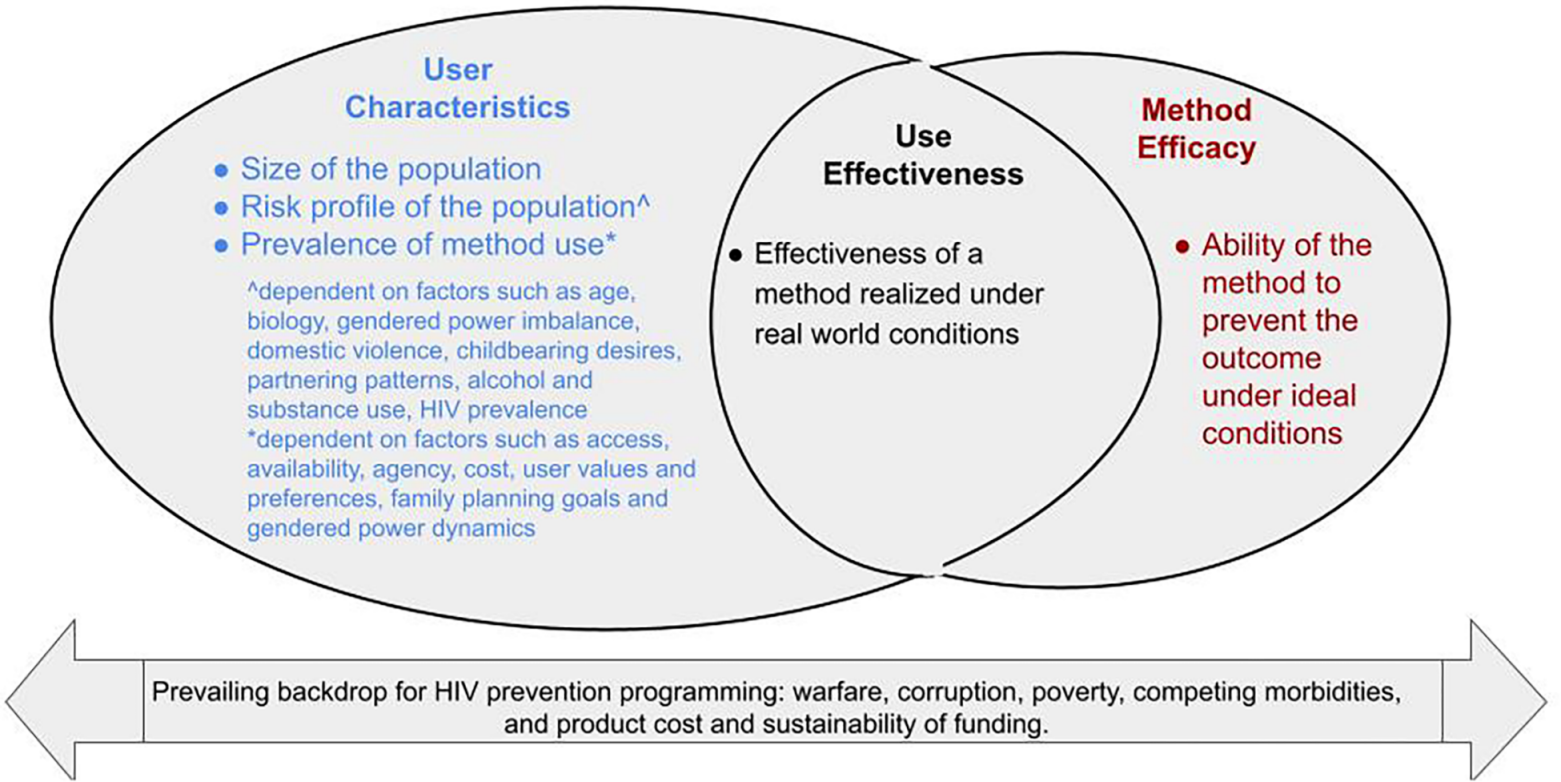
Drivers of population benefit.

With respect to factors that public health practitioners can influence, drivers of population benefit involve the interplay of three broad elements (Figure 1). First are the population's size, risk profile (whether the group is epidemiologically at high risk for HIV and the total fertility rate), and prevalence of preventive method use in at-risk populations. Second is method efficacy which is the method's ability to prevent the outcome under ideal conditions, such as efficacy observed during pre-clinical challenges or in as-treated analyses in efficacy trials when a method is used consistently and correctly (8). Third, is use-effectiveness which is the effectiveness of a method realized under real-world conditions, where factors such as uptake and consistent and correct use influence the ultimate protection conferred (8).

A highly efficacious method only provides benefit if used consistently and correctly. A widely-used prevention method with only moderate method efficacy may still have population impact as was seen with the use of withdrawal (coitus interruptus) for contraception that contributed to the demographic shift in family size at the end of the 19th century (9). The interplay of method efficacy and user adherence, an element determining use-effectiveness, is demonstrated by the mixed findings of oral PrEP (TDF/FTC) to prevent HIV among women (10). High method efficacy alone is not sufficient to shift an epidemic. The role of the user, their risk profile, their access to methods, and ability to consistently and correctly use a method, are important drivers of protection. Long-acting cabotegravir (CAB-LA) has demonstrated high method efficacy for HIV prevention in clinical trial settings (11). Method efficacy for CAB- LA is derived from select women who met inclusion criteria, received reimbursements for study visits, and were cared for by proactive staff reminding them of injection visits. While many women in SSA state preferences for injectable HIV prevention (2), the use-effectiveness of CAB-LA to protect women from HIV under real-world scale-up is yet to be characterized. Further, at a population level, if new CAB-LA users are those migrating from successful daily oral PrEP use, population impacts on the HIV epidemic may be moderate if one method is simply supplanted for another without a net gain in prevention coverage for those at risk for HIV. The Catalyzing Access to New Prevention Products to Stop HIV (CATALYST) study, will evaluate a multi-product service-delivery platform offering daily oral PrEP, the monthly dapivirine ring, and CAB-LA at public clinics across five countries in southern and eastern Africa and will provide data on use-effectiveness of these single-indication methods and describe the programmatic impact of offering choice (12).

## Three drivers + a MPT for HIV and pregnancy

Adding a MPT for HIV and pregnancy to this three-part equation may result in beneficial scenarios by adding new users with unmet need for both indications resulting in a net increase in population coverage, or by expanding protection for a second indication among existing users with unmet need. Increasing uptake for new users holds promise as MPTs are highly desired with 96% of women surveyed in SSA preferring HIV prevention products with multiple indications compared to a single-indication product (2). Preference for multiple over a single indication outweighed preference for type of delivery method (i.e., injectable, pill, implant, etc.) (2). Further, in a worldwide survey of method preference, women were most interested in products offering both HIV and pregnancy prevention (82%) compared with single indications for HIV (76%) or pregnancy only (64%) (13).

In both scenarios above–either attracting new users or expanding coverage to those with unmet need for a second indication–additional benefit is realized so long as the added coverage occurs among women at high risk for the outcomes as suggested by cost effectiveness modeling. Analyses scaling a MPT for HIV and pregnancy found pregnancy prevention would be cost effective if rolled out to women at high risk of HIV (e.g., serodiscordant couples, AGYW or female sex workers) but not to a general population of women (14,15). Notable was that a MPT for HIV and pregnancy was cost effective among populations at high risk for HIV even with HIV method efficacy ranging from 45% to 75% (15). Modeling of the dual prevention pill (DPP) found that scaling the DPP was sensitive to both risk of HIV in the population and adherence (e.g., use-effectiveness), where if adherence was low, the health risks from unintended pregnancy could outweigh the health benefit of HIV prevention (14).

## MPT potential benefits and pitfalls for individual users and public health

Expanding protection for the dual outcomes of HIV and unplanned pregnancy would be especially beneficial among AGYW in SSA who are disproportionately at risk for HIV, have unmet contraceptive needs, and face well-established barriers to accessing sexual and reproductive health services (16). The availability of a MPT may ease how AGYW interface with the healthcare system by meeting dual health care needs with only one health encounter which may be less stigmatizing if focused on reproductive health compared with HIV prevention, and by reducing multiple disclosures about HIV and sexual activity (2, 17). Additionally, use-effectiveness for a HIV and pregnancy MPT may be enhanced if women are highly motivated to use the method consistently and correctly in order to avoid unplanned pregnancy as self-perceived risk for HIV is often inaccurate (18,19). However, there are potential pitfalls for a MPT highly dependent on adherence, such as the daily oral DPP. As per DPP cost-effectiveness modeling, expanded population coverage achieved could be outweighed by poor adherence and lowered use-effectiveness, as daily pill taking may be more challenging than using longer-acting methods (14). Since the average probability of conception is higher than HIV transmission, poor adherence to the daily oral DPP could be offset by unplanned pregnancy without necessarily increasing HIV protection.

The introduction of a MPT for HIV and pregnancy, especially among AGYW in SSA, has the potential to offer health dividends not just for the individual user by reducing HIV incidence and unplanned pregnancy in adolescence, but may also improve maternal and child health outcomes. Pregnancy in adolescent girls is associated with disproportionately high maternal mortality, low infant birth weight, severe neonatal outcomes, as well as decreased education and censored socioeconomic potential impacting the welfare of both mother and child (20).

A MPT for HIV and pregnancy may have the additive effect of streamlining and integrating service delivery for over-burdened providers and public health systems, a need which has long been noted (21–23). Most family planning and HIV clinics are overburdened and may not have capacity to provide separate but overlapping services yet several models for integrated care have been proposed (24,25). Integrated service delivery settings are associated with increased method uptake, enhanced client satisfaction, reduced HIV-related stigma, and may facilitate the involvement of men and improve joint decision making around protection (21, 23, 26). Yet, the development of a biomedical MPT for HIV and pregnancy will be challenging, as it requires meeting safety and efficacy thresholds for two indications while regulatory standards for a dual-indication product remain unclear (1, 27).

## Discussion

Adolescent girls and young women who bear a disproportionate burden of HIV and unplanned pregnancy have much to gain from a MPT for HIV and pregnancy. While such MPTs are highly desired and have considerable potential for public health impact, like single-indication products, there is no single MPT, regardless of method efficacy level, that alone, will stem the twin epidemics of HIV and unplanned pregnancy. The most effective method is the one that gets used consistently and correctly. While continued efforts should focus on developing MPTs offering high method efficacy for intended indications, a MPT for pregnancy and HIV, even with limited method efficacy, has the potential to have significant epidemic impact if used among those at high risk for HIV. Further, women need a range of prevention methods that can be varied across their lifetime, given that values, preferences, needs and risks vary across the life course. The contraceptive field has shown that offering a choice of methods, even with a range of method efficacy, lessens the burden of unmet need for family planning because values and preferences of users are varied (28). Contraceptive method decision-making is influenced by a variety of factors beyond efficacy including reproductive health events, relationship status, partner approval, childbearing desires, and societal norms which all affect the ultimate ability to adopt and use a method. Key programmatic features that would maximize the potential of MPTs include offering MPT as part of a range of safe and effective contraceptive and HIV prevention methods with full information about their features such as indications addressed (single, dual, multiple), method efficacy, use-effectiveness, mechanism of action, potential side effects, return to fertility, duration, and respectful support in how to use. This would allow women to make a truly informed method choice. Offering an array of both single and dual methods for contraception and HIV and ensuring providers have strong integrated counseling guidelines and training may mitigate against pitfalls, such as inducing women to switch to a less-effective MPT. A method array that includes multiple and single indications, locally-acting and systemic products, as well as on-demand, medium-, and long-acting methods is necessary to address women's complex and evolving needs.

USAID is investing in the development of an array of biomedical HIV prevention products for AGYW, and a few of these products are being developed as MPTs for HIV and pregnancy. Additionally, many USAID service delivery initiatives are underway to streamline and decentralize HIV services in high HIV-burden countries. In the short term, USAID supports moving to integrated care models for sexual and reproductive health, including moving more services into primary care, which is important for sustaining and simplifying service delivery. In the absence of MPTs available for implementation, additional testing of co-delivery models providing existing HIV and pregnancy prevention methods (e.g., the administration of injectable CAB-LA along with contraception) are needed given the promising association that integrated service delivery has on dual method use. In the longer term, sustained investments to make safe, effective, acceptable, and scalable MPTs a reality should continue.

## Data Availability

The original contributions are included in the article. Further inquires can be directed to the corresponding author.
